# Unearthing the Root of Amino Acid Similarity

**DOI:** 10.1007/s00239-013-9565-0

**Published:** 2013-10-01

**Authors:** James D. Stephenson, Stephen J. Freeland

**Affiliations:** NASA Astrobiology Institute, University of Hawaii, Honolulu, HI 96822 USA

**Keywords:** Amino acids, Simplified alphabets, Similarity measures, Chemical properties, Protein structure

## Abstract

Similarities and differences between amino acids define the rates at which they substitute for one another within protein sequences and the patterns by which these sequences form protein structures. However, there exist many ways to measure similarity, whether one considers the molecular attributes of individual amino acids, the roles that they play within proteins, or some nuanced contribution of each. One popular approach to representing these relationships is to divide the 20 amino acids of the standard genetic code into groups, thereby forming a simplified amino acid alphabet. Here, we develop a method to compare or combine different simplified alphabets, and apply it to 34 simplified alphabets from the scientific literature. We use this method to show that while different suggestions vary and agree in non-intuitive ways, they combine to reveal a consensus view of amino acid similarity that is clearly rooted in physico-chemistry.

**Electronic supplementary material:**

The online version of this article (doi:10.1007/s00239-013-9565-0) contains supplementary material, which is available to authorized users.

## Introduction

The relationships between the 20 amino acids of the standard genetic code are fundamentally important to the folding (Haber and Anfinsen 1962), interactions (Lucchese et al. 2012), and evolution (Dayhoff et al. 1978) of proteins. One approach to understanding these relationships is to create a simplified amino acid alphabet by grouping the full set of 20 according to their similarity (for example, a scheme that separates hydrophobic from hydrophilic amino acids has simplified the alphabet from size 20 to 2). The publication of many different simplified alphabets demonstrates the usefulness of this approach. Such simplified alphabets can improve topological estimation in phylogenetic analysis (Susko and Roger 2007) and aid in the classification of proteins (Albayrak et al. 2010; Chen et al. 2012). An appropriately simplified alphabet of size 12 has also been shown to exhibit better selectivity and sensitivity to predicting protein folds than the full set of 20 (Peterson et al. 2009).

This range of practical uses has led amino acid similarity to be defined in many ways such that simplified alphabets may reflect the purpose for which they were constructed or the methods by which they were derived. For example, similarity measurements which focus on the chemistry and physics of individual amino acid molecules (Mahler and Cordes 1966; Lehninger 1970; Dickerson and Geis 1983; Taylor 1986; Weathers et al. 2004) are likely to be different from those which analyze the roles played by amino acid residues within protein sequences (e.g., Dayhoff et al. 1978; Risler et al. 1988; Riddle et al. 1997; Murphy et al. 2000; Etchebest et al. 2007) simply because biology’s genetic code defines how many point mutations are required to interconvert two different amino acids during protein sequence evolution (Fitch 1966). This effect, which has been reported and estimated from biological sequence data (Benner et al. 1994; Yampolsky and Stolzfus 2005; Di Giulio 2008), has no counterpart within the chemistry of individual amino acid molecules. Furthermore, specific amino acids can play special roles within the proteins in which they occur. Cysteine, for example, pairs with itself to form disulfide bridges that stabilize protein structures (Haber and Anfinsen 1962), and proline’s unique structure causes natural selection to favor its use in proteins for interrupting alpha helices. These behaviors are difficult to identify, without the benefit of hindsight, when considering only the chemical properties of a single amino acid molecule. Broadening this view, it has been estimated that around 35 % of the genetically encoded amino acids’ potential to form secondary structures comes from non-local molecular interactions (Gu and Bourne 2009). Such interactions can be more conserved within structural evolution than the identity of specific amino acid residues (Noivirt-Brik et al. 2013). Most generally of all, any specific effort to calculate an amino acid simplification scheme will contain some degree of experimental error.

To investigate these and other possible causes of agreement and disagreement between different simplified alphabets, we have developed a new method to compare and combine two or more simplified amino acid alphabets. Using this framework, we have investigated how different simplified alphabets vary from one another and then considered whether a consensus view of the different alphabets provides a meaningful, global measure of amino acid similarity.

## Results

### Comparing Simplified Amino Acid Alphabets

In order to investigate the variability between different simplified alphabets, we identified a comprehensive dataset consisting of 34 amino acid simplifications published within peer-reviewed literature and characterized them according to their method of derivation (Table 1). Within each simplified alphabet, amino acids are grouped together on the basis of similarity such that each amino acid belongs to exactly one group. An alphabet can therefore range in size from 1 (for an approach that accepts all 20 amino acids as similar enough to be considered equivalent) to 20 (where the discrimination is so fine-grained that no 2 amino acids are considered similar enough to be considered equivalent).

**Table 1:** Details of 34 simplified alphabets taken from peer reviewed scientific literature

**a** In each case, the alphabet size was chosen according to that deemed best by the source study in question. Within each simplified alphabet, amino acid groupings are comma delimited. Branch score distances indicate the effect of dropping each study on the overall consensus tree (Fig. 3); **b** Different simplified alphabets are color coded according to the methods by which each was derived (not according to the theme of the source manuscript)

**Fig. 1:**
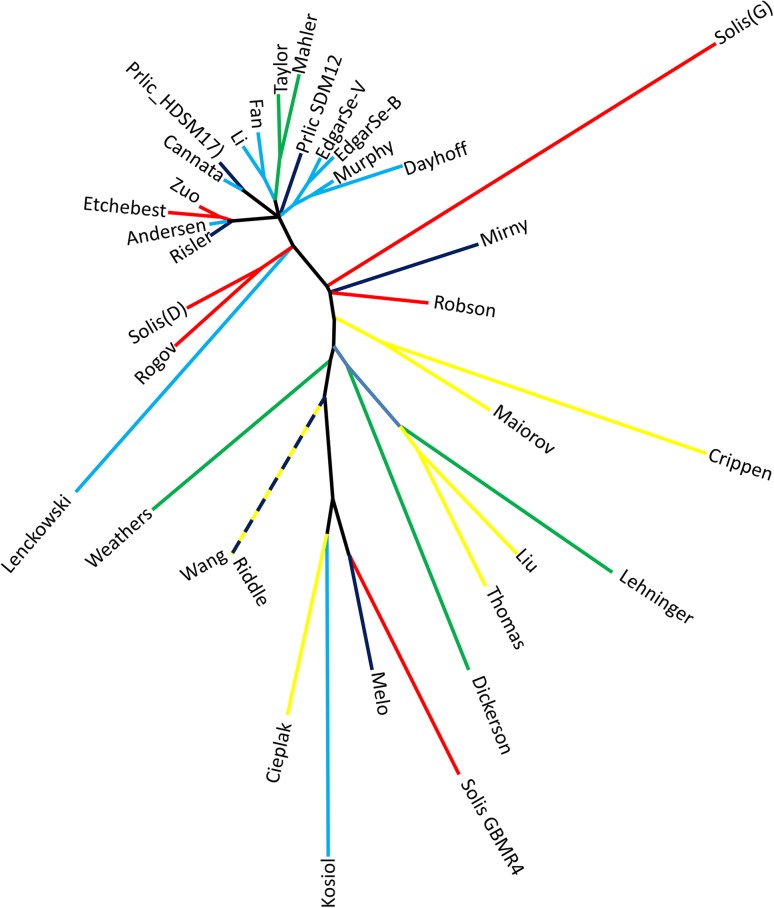
Simplified amino acid alphabets colored according to the method by which they were derived. Dendrogram derived by least squares from the relative similarities of 34 published simplified amino acid alphabets, labeled by Stephenson.* Longer branch lengths* indicate lower similarity between two alphabets;* colors* represent method by which each simplified alphabet was derived as described in Table 1

Converting each alphabet into an appropriate matrix format (see “Methods”) enabled an all-against-all pairwise comparison of the 34 different simplification schemes. From these comparisons, a new matrix was created to record the proportion of amino acid pairs grouped in the same way in both simplification schemes for all possible pairs (Supplementary Table S1). Figure 1 shows a least squares derived dendrogram of this similarity matrix, colored according to the method by which each simplified alphabet was derived.

This analysis demonstrates that disparate derivation techniques can result in similar simplified alphabets and vice versa. In particular, Fig. 1 shows that approaches which consider physico-chemical properties of individual amino acids are often found close to those which infer amino acid similarity from analysis of protein sequence or structure information.

**Fig. 2:**
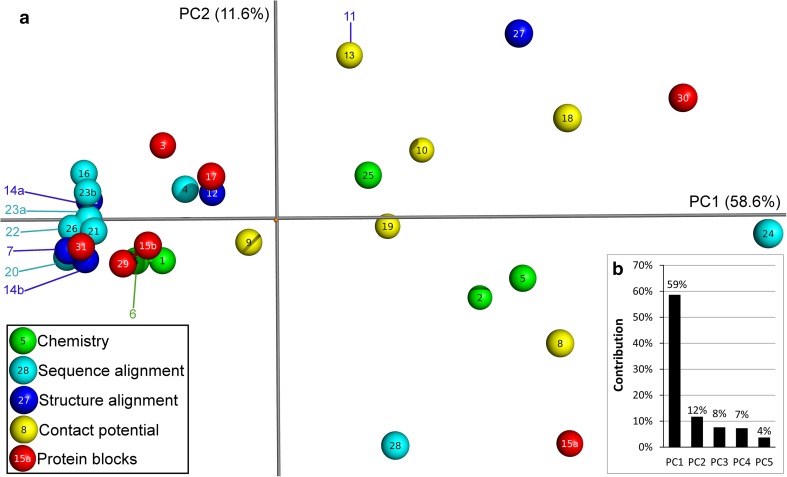
Principal components 1 and 2 of the 34 × 34 simplified alphabet similarity matrix colored by derivation method. **a** Simplified alphabets are shown as *spheres* and labeled according to the alphabet ID numbering in Table 1. **b** Variance contribution of the first five principal components of this analysis

In order to further investigate the variation within and between simplified alphabets, the 34 × 34 alphabet comparison matrix (Supplementary Table S1) was reduced by principal components analysis (Fig. 2).

Several patterns are evident in Fig. 2. For example, structure (Risler et al. 1988; Riddle et al. 1997; Mirny and Shakhnovich 1999; Prlic et al. 2000; Melo and Marti-Renom 2006) and sequence (Dayhoff et al. 1978; Murphy et al. 2000; Cannata et al. 2002; Fan and Wang 2003; Li et al. 2003; Edgar 2004; Kosiol et al. 2004; Andersen and Brunak 2004; Lenckowski and Walczak 2007) alignments generally group together (although both have distant outliers); most simplified alphabets based upon protein blocks (Robson and Suzuki 1976; Solis and Rackovsky 2000; Rogov and Nekrasov 2001; Etchebest et al. 2007; Peterson et al. 2009; Zuo and Li 2009) also associate here. Simplifications based on contact potentials (Crippen 1991; Maiorov and Crippen 1992; Thomas and Dill 1996; Wang and Wang 1999; Cieplak et al. 2001; Liu et al. 2002) are distributed more evenly, comprise fewer outliers, and do not cluster with the alignment methods. This suggests that considering amino acid similarity in terms of contact potentials offers a genuinely fresh perspective. Simplification schemes based on chemistry (Mahler and Cordes 1966; Lehninger 1970; Dickerson and Geis 1983; Taylor 1986; Weathers et al. 2004) straddle contact potentials and the main alignment cluster. The mean Cartesian value of all simplified alphabets on the first two principal components plane is (0,0). The PC2 axis partitions most of the alphabets derived from contact potentials from those derived from alignments. Interestingly, the furthest outliers from this main cluster (Solis and Rackovsky 2000; Kosiol et al. 2004; Melo and Marti-Renom 2006; Lenckowski and Walczak 2007; Peterson et al. 2009) are dominated by later additions to the literature. This may be because they represent new ways of clustering or that they represent significant improvements for a given task which occupies a distinct area of solution space. It is important to recognize that the relative position of a study reveals nothing about its quality; indeed we encourage future work to investigate relationships between position and function of a simplified alphabet.

For a more complete understanding of the relationships between studies, Fig. 2 should be considered alongside Fig. 1. Both representations have necessarily lost information from the matrix comparisons to allow the data to be visualized. For example, principal components 1 and 2 account for only 70.2 % of the variation between studies.

### Comparing Consensus Views

**Fig. 3:**
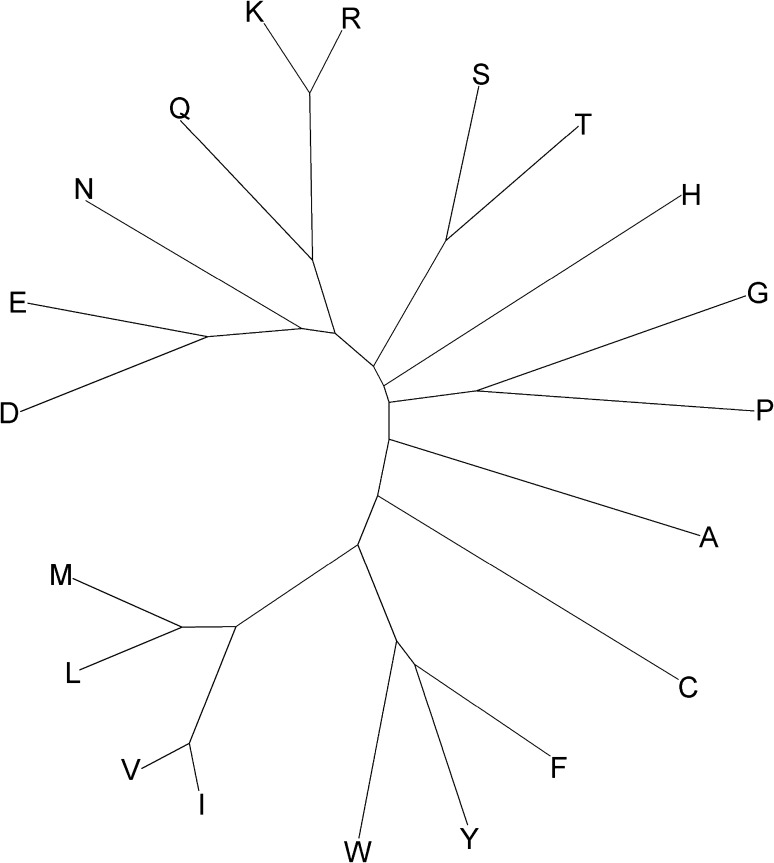
Consensus amino acid similarity dendrogram from 34 alphabets. Dendrogram constructed by least squares using the similarity data from all 34 simplified amino acid alphabets.* Long branches* indicate that an amino acid is rarely grouped with any other as part of a simplification scheme. Short path lengths between amino acids suggest high similarity between them

In order to further investigate the effects of derivation method on amino acid similarity, the 34 individual simplification schemes were combined (see “Methods”) to create a consensus tree view of amino acid similarity rather than of alphabet similarity (Fig. 3).

**Fig. 4:**
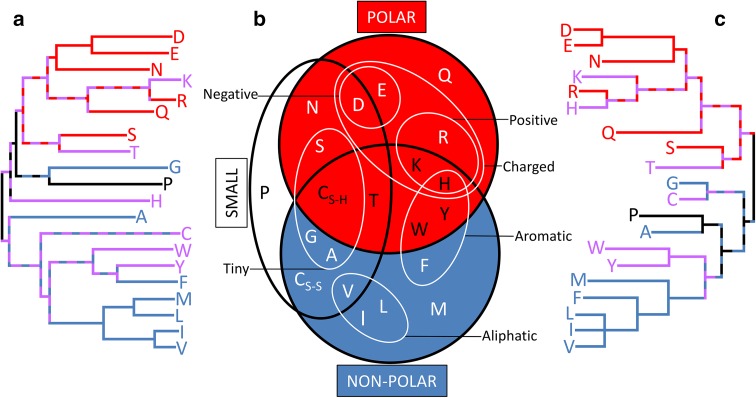
Amino acid similarity relationships defined by analysis of proteins closely resemble those derived from analysis of individual amino acid chemistry. Dendrograms constructed by least squares using the similarity data from **a** 29 studies which considered amino acid residues within proteins sequences and structures, versus **c** 5 simplified alphabets which were derived from individual amino acid physico-chemistry.* Long branches* indicate that an amino acid is rarely grouped with any other as part of a simplification scheme. Short path lengths between amino acids suggest high similarity between them. Comparing both dendrograms with a redrawn version of a commonly used chemical property Venn diagram **b** adapted from Livingstone and Barton (1993) uncovers the physico-chemical basis for many of the dendrogram features. The hydrophobic (*blue*), polar (*red*), and both hydrophobic and polar (*purple*) amino acids are colored to highlight this principal basis of organization within each of the dendrograms

The robustness of the tree was checked by a jack-knife approach which showed that no single scheme had a significant effect on tree structure (final column in Table 1). Several amino acids appear to be ungrouped in the dendrogram, and each instance demands a separate explanation. Histidine (H) for example is likely grouped differently in different schemes due to its versatility in proteins. The multiple roles it plays in protein interactions have caused it to be labeled the most active member of the amino acids (Liao et al. 2013). Cysteine (C) perhaps plays one of the most vital roles in influencing 3D folding of proteins via disulfide bond formation (Muskal et al. 1990). However, it plays a different role when not bonded with another cysteine which is why it has two entries (C_S–S_ and C_S–H_) in the chemistry Venn diagram shown in Fig. 4b. Alanine (A) is not very hydrophobic and is non-polar; it is therefore present in many non-critical protein contexts (Betts and Russell 2003). Because it can appropriately be clustered in several ways, there is little consensus as to its closest amino acid neighbors.

By grouping the most similar amino acids from the dendrogram in Fig. 3 hierarchically, a simplified amino acid alphabet from size 19 to 2 could be defined. However, as discussed later, more information is maintained by presenting the pairwise matrix than any single alphabet. Also, the ideal size of the simplified alphabet may depend on the task for which it will be used.

The 34 simplified alphabets shown in Fig. 1 and Table 1 were separated into 2 groups according to whether they were derived from consideration of amino acid residues within proteins, or from consideration of the physico-chemical properties of individual amino acids (Table 2). Data in Table 2a,c were then used to construct dendrograms (Fig. 4), which suggest a striking agreement between each view of amino acid similarity.

**Table 2:** Consensus distance matrices when considering **a** amino acids as residues within proteins; **b** physico-chemical properties of individual amino acids; and **c** the difference between these two approaches

The values shown in **a** and **b** are distances between amino acids, calculated by subtracting the similarity measurements from 1. A value of 0 indicates that an amino acid pair is always grouped together in all simplified alphabets; conversely, a value of 1 indicates that the pair is never grouped together. The absolute differences between each cell in **a** and **b** are shown in **c**. All matrices are colored using the same scale from 0 (*green*) to 1 (*red*)

The dendrogram similarity is noteworthy in that Fig. 4c may be considered the result of dropping 29 studies: in other words, the underlying picture of amino acid similarity is sufficiently clear and robust that just 5 independent studies which focus upon the physico-chemical properties of amino acids find essentially the same picture as (i) 29 studies which focus on protein sequences and (ii) all 34 studies combined (Fig. 3).

This unified picture of amino acid similarity is particularly clear when both dendrograms are compared with Fig. 4b, a redrawn version of a widely promoted visualization of amino acid chemistries (Livingstone and Barton 1993). For example, hydrophobicity and/or polarity appear to define the principal dimension of chemical similarity (corresponding to the deepest branch points on dendrograms in Fig. 4a, c). No hydrophobic (blue) amino acid is ever clustered with a polar (red) amino acid and vice versa, whereas the scattered distribution of differently sized amino acids throughout the dendrogram suggests that size is a less important measure. Beyond this deepest level of similarity, several other clusters within the dendrograms, such as the distribution of positively charged amino acids, match those found in the Venn diagram. At an even finer level of inspection, the members of each amino acid pair considered similar (grouped) by the majority of the studies (Table 2) are always found in close proximity in the chemistry space Venn diagram (Fig. 4b).

Although this pattern of agreement might be anticipated between Fig. 4b and Fig. 4c in as much as the Venn diagram (Fig. 4b) is itself derived by from consideration of amino acid chemistry, it is more surprising for Fig. 4a which was built exclusively from data regarding amino acid moieties within the context of protein sequences and structures, unconcerned with individual amino acid properties.

To quantify the agreement between Fig. 4a and Fig. 4c, it is tempting to measure directly the similarity of the dendrograms; however, two reasons argue against this approach. First, any comparison of dendrograms is a subtle challenge that remains an area of active research (Morlini and Zani 2012). Second and more fundamental, while these dendrograms are useful for visualizing the complex web of relationships between the set of amino acids (compare Fig. 4 with Table 2), they are derived from information-rich matrices that record independent estimates of the distance between every amino acid and all others. Much of this detailed information is lost in the process of translating the matrix into its corresponding dendrogram. A more accurate and straightforward quantification of the similarity therefore comes from measuring the similarity of the two matrices from which Fig. 4a, c was derived.

**Fig. 5:**
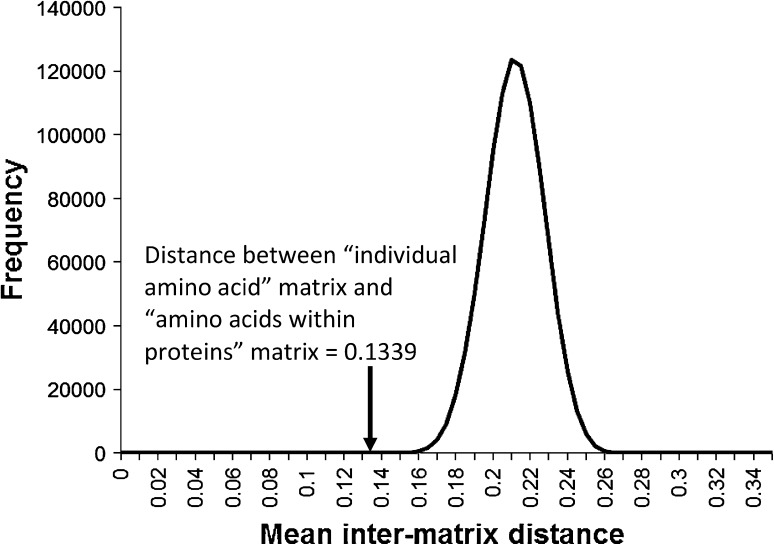
Distance between matrices when considering amino acids within proteins and when considering their individual amino acid physico-chemical properties against a background of randomized matrices. Frequency distribution of inter matrix distances between the “individual chemistry” matrix calculated in this study and 1,000,000 random matrices (randomizing rows only) generated from real matrix seeds. The distance between the two matrices (Table 2a, b) was 0.1339

Figure 5 shows the difference between these distance matrices alongside a corresponding distribution of differences for a large sample of alternative randomized matrices (Mantel test Mantel 1967).

The analysis illustrated in Fig. 5, based on total information regarding amino acid similarity, reveals a vanishingly small probability that the overall degree of similarity seen in Fig. 4 could occur by chance.

## Discussion

Here, we have introduced a simple new method to compare and combine different suggestions for grouping the 20 amino acids of the standard genetic code into simplified alphabets. On the one hand, this method shows how individual simplification schemes vary from one another, providing a single visualization (Fig. 2) that summarizes three decades of research. On the other hand, it reveals that a composite view of all simplified alphabets reflects a view of amino acid similarity firmly rooted in physico-chemistry.

Both of these observations offer useful platforms for further research. Although previous studies have measured how different simplified alphabets perform relative to one another in terms of a specific bioinformatics challenge (e.g., Dosztanyi and Torda 2001), our method measures different simplified alphabets relative to one another, independent of any particular application. This reveals patterns of variability that were not intuitive (including unexpected agreements between different approaches, and outliers within a single approach) and thus provides a context for future contributions to the scientific literature of simplified alphabets. Since bioinformatics tools may rely on which simplified alphabet is used, it will be interesting to see whether specific regions of Fig. 2 perform better at specific tasks. This in turn would advance meaningful classification and rational design of simplified alphabets.

More surprising to us is that a composite of 34 diverse simplified alphabets converges upon a view of amino acid similarity that can be explained directly in terms of physico-chemistry. Given that only 5 out of 34 schemes directly identify themselves as measuring physico-chemical properties of amino acids, this result was not a foregone conclusion. It may be intuitive that physico-chemical properties underlie sequence evolution and protein structure but other factors, such as the organization of the genetic code, the peculiar roles played by specific amino acids in specific proteins, and an unknown degree of experimental error in the derivation of each simplified alphabet could have combined to obscure any underlying agreement. Instead we observe a *vox populi* effect (Galton 1907) whereby experimental error, individual differences and systematic biases that divide different approaches to generating a simplified alphabet cancel each other out.

The significance of this latter point is that until now, most insights regarding amino acid similarity have come from observations of the 20 amino acids of the standard genetic code. However, two additional amino acids (selenocysteine and pyrrolysine) occur in non-standard codes, and many others result from posttranslational modifications and non-ribosomal peptide synthesis. Connecting the behavior of amino acids within proteins with physico-chemical properties that can be measured for any amino acid molecule forms an important step toward broadening biological theory to encompass amino acids beyond those found in the standard genetic code.

## Methods

### Binary Intra Group Matrices

**Fig. 6:**
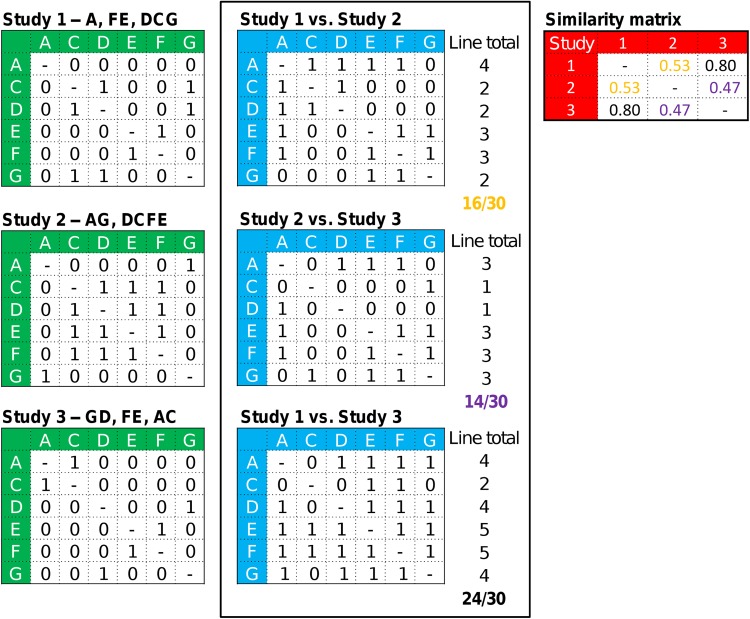
Illustration of the method used to compare simplified amino acid alphabets using a fictional 6-letter alphabet for clarity of example. The groupings described by three simplifications, named studies 1–3, for a fictional 6-letter alphabet are initially described as comma-delimited text (shown above each of the *green* matrices, *left*). The contents of the *green* matrices thus represent each simplified alphabet: within each matrix, a value of 1 indicates that two amino acids are grouped as “similar”; a value of 0 indicates otherwise. The* blue* matrices are constructed by comparing each element in the* green* matrices pairwise. This time, a match between the corresponding cells for two* green* matrices results in a 1 within the *blue* matrix (0 represents a mismatch). Summing the matched values from the *blue* matrices results forms an overall similarity value, as shown in the final rows of the “line total” column. These similarity values can be assembled in a similarity matrix, shown in *red*, which records all pairwise inter-alphabet similarities. In this example, alphabets from studies 1 and 3 are the most similar and from 2 and 3 are the least similar

We collated all published simplification schemes that highlight 1, specific simplified alphabet as the most relevant to the task at hand. Converting comma-delimited simplified alphabets (Table 1) into a uniform, matrix representation reduced the challenge of comparing or combining different suggested simplified alphabets into a straightforward manipulation of their corresponding matrices. The first step toward this goal was to construct what we term a ‘Binary Intra Group’ (B.I.G) matrix, of 20 × 20 elements in which to record similarity between every possible pairing of the 20 amino acids according to a specific simplified alphabet. Within this B.I.G matrix, elements were set to 1 wherever a grouping scheme reported two amino acids as being similar (grouped together), and 0 otherwise (green matrices, left most column in Fig. 6).

For example, a scheme which reports aspartic acid (D) and glycine (G) as similar to each other but both different from tryptophan (W) would record matrix elements [D, G], [G, D], [G, G], [D, D], and [W, W] as 1 and all other matrix elements as 0. Since the main diagonal of the matrix reflects whether an amino acid groups with itself (always true) or not (never true), it was ignored. All 34 amino acid simplification suggestions (Table 1) were converted into B.I.G matrices (Perl script available from the corresponding author).

### Comparing Simplified Alphabets

Our comparison of simplified alphabets began with the construction of a second (empty) matrix. In this new matrix, elements were set to 1 wherever the corresponding elements of two B.I.G matrices matched and 0 elsewhere (blue matrices, central column in Fig. 6): note, as before, the main diagonal of this matrix is without information.

The similarity between two simplified alphabets was estimated by summing the elements of the inter-alphabet comparison matrix and dividing by the maximum total possible (=380) to produce a single value that ranges from 0 to 1 to indicate the similarity of the two suggestions (1 indicates identical suggestions, and 0 indicates that a pair of simplified alphabets share nothing in common). The comparison values were then collated into a similarity matrix (Perl script available from the corresponding author), showing the similarity between any pair of alphabets (red matrix, rightmost column in Fig. 6).

Alphabet simplifications were then characterized according to the methods by which they were derived (Fig. 1). In particular, we distinguished approaches which consider the physico-chemical properties of individual amino acids from approaches which derive from analysis of amino acid moieties within proteins. These latter studies were further sub divided as shown in Table 1b. The distance table (Supplementary Table S1) was used to make a dendrogram by neighbor joining followed by least squares refinement using MEGA v5.05 (Tamura et al. 2011), with individual branches colored according the derivation categories shown in Table 1b. The topology of the dendrogram was unchanged when using neighbor joining, minimum evolution, or least squares. Our choice of methods for deriving this dendrogram reflects the simplicity of our data type (no underlying state-types). In particular, we avoided the use of other tree-generation methods that would introduce inappropriate assumptions for our data (for example, unweighted pair group mean average assumes equal rates of evolution across branches and builds a rooted tree. These concepts are logically inconsistent with our analysis, as we posit no evolutionary ancestry within the amino acids).

### Combining Simplified Alphabets

The data from all simplified alphabets were combined by adding the numbers at each amino acid pair position (each cell) from the 34 B.I.G tables and entering them into a new 20 × 20 B.I.G consensus matrix. The scores of each element (the number of studies which suggested that the amino acid pair should be grouped) were divided by the total possible score (34) to calculate an amino acid similarity consensus matrix (Perl script available from the corresponding author). This B.I.G consensus matrix was used to construct a dendrogram (Fig. 3) in MEGA v5.05 software (Tamura et al. 2011) using neighbor joining followed by least squares refinement. Thirty-four new dendrograms were constructed by jack-knifing the data (i.e., systematically dropping one study at a time). The resulting 34 new trees were compared to the original study (built from all 34 studies) using the branch score distance method (Kuhner and Felsenstein 1994) as implemented in Phylip. Branch score distances are provided in the final column of Table 1.

The data used to construct the consensus tree in Fig. 3 were split into categories (Table 2a, b) and used to make comparable dendrograms (Fig. 4a, c). (The topology of the dendrogram was unchanged when using neighbor joining, minimum evolution, or least squares.) Livingstone and Barton’s (1993) widely used Venn diagram of amino acid similarity was redrawn as Fig. 4b to facilitate visual comparisons between dendrograms.

### Quantifying Tree Similarity

The sum of the absolute distances between corresponding cells of the separated matrices Table 2c was used to quantify the two groups of simplified alphabets visualized in Fig. 4a, c. This summed distance was then calculated between our “individual amino acid chemistry” distance matrix and 1,000,000 randomizations of the “amino acids within proteins” matrix and also vice versa (i.e., our “amino acids within proteins” distance matrix and 1,000,000 randomizations of the “individual amino acid chemistry” matrix). For each test, randomized matrices were constructed by randomizing the row order of the compared matrix. Only the rows were randomized, as randomizing both columns and rows would likely inflate the perception of similarity between our “individual amino acid chemistry” and “amino acids within proteins” pictures of amino acid similarity (because significant, structured information is present within each column or row). Our more conservative randomization is therefore more sensitive to recording whether these two views of amino acid similarity genuinely concur. To check whether the random matrices were representative of the possible solution space we ran 10 tests scoring the distances between “individual amino acid” matrix and 100,000 random matrices and 10 tests of the distances between “within protein” matrix and 100,000 random matrices. We found that the 20 group means of distances differ little from each other 0.2094–0.2095. The range of all values was 0.164–0.253 (3SF). This suggests that we have adequately sampled the possibility space of random matrices and that the distance between “individual amino” acid and “within proteins” (0.1339) is exceptional. While this may be considered a small sample of the 20! (2.4 × 10^18^) ways of ordering 20 objects, the degree of variation seen here gives us confidence in these results.

Even here, the results should be treated with care. As seen in the visualization shown in Fig. 4, there are differences in the details (e.g., as anticipated, histidine and cysteine appear somewhat different according to whether a simplified alphabet is derived from analysis of proteins or a consideration of physico-chemical properties): our analysis therefore demonstrates only that the overall perception of amino acid similarity derived from these two different approaches is indeed highly convergent.

## Electronic Supplementary Material

Below is the link to the electronic supplementary material.

Supplementary material 1 (PDF 236 kb)

## References

[CR1] Albayrak A, Out HH, Sezerman UO (2010). Clustering of protein families into functional subtypes using relative complexity measure with reduced amino acid alphabets. BMC Bioinformatics.

[CR2] Andersen CAF, Brunak S (2004). Representation of protein-sequence information by amino acid subalphabets. AI Magazine.

[CR3] Benner SA, Cohen MA, Gonnet GH (1994). Amino acid substitution during functionally divergent evolution of protein sequences. Protein Eng.

[CR4] Betts MJ, Russell RB (2003). Amino acid properties and consequences of substitutions. Bioinformatics for geneticists.

[CR5] Cannata N, Toppo S, Romualdi C, Valle G (2002). Simplifying amino acid alphabets by means of a branch and bound algorithm and substitution matrices. Bioinformatics.

[CR6] Chen W, Feng P, Lin H (2012). Prediction of ketoacyl synthase family using reduced amino acid alphabets. J Ind Microbiol Biotechnol.

[CR7] Cieplak M, Holter NS, Maritan A, Banavar JR (2001). Amino acid classes and the protein folding problem. J Chem Phys.

[CR8] Crippen GM (1991). Prediction of protein folding from amino acid sequence over discrete conformation spaces. Biochemistry.

[CR9] Dayhoff MO, Schwartz RM, Orcutt BC (1978) A model of evolutionary change in proteins. Atlas of protein sequence and structure, National Biomedical Research Foundation, p 345–351

[CR10] Di Giulio M (2008). The origin of the genetic code cannot be studied using measurements based on the PAM matrix because this matrix reflects the code itself, making any such analyses tautologous. J Theor Biol.

[CR11] Dickerson RE, Geis I (1983). Hemoglobin: structure, function, evolution, and pathology.

[CR12] Dosztanyi Z, Torda AE (2001). Amino acid similarity matrices based on force fields. Bioinformatics.

[CR13] Edgar RC (2004). Local homology recognition and distance measures in linear time using compressed amino acid alphabets. Nucleic Acids Res.

[CR14] Etchebest C, Benros C, Bornot A, Camproux AC, de Brevern AG (2007). A reduced amino acid alphabet for understanding and designing protein adaptation to mutation. Eur Biophys J.

[CR15] Fan K, Wang W (2003). What is the minimum number of letters required to fold a protein?. J Mol Biol.

[CR16] Fitch WM (1966). An improved method for testing for evolutionary homology. J Mol Biol.

[CR17] Galton F (1907). Vox populi. Nature.

[CR18] Gu J, Bourne PE (2009). Structural bioinformatics.

[CR19] Haber E, Anfinsen CB (1962). Side-chain interactions governing the pairing of half-cystine residues in ribonuclease. J Biol Chem.

[CR20] Kosiol C, Goldman N, Buttimore NH (2004). A new criterion and method for amino acid classification. J Theor Biol.

[CR21] Kuhner MK, Felsenstein J (1994). A simulation comparison of phylogeny algorithms under equal and unequal evolutionary rates. Mol Biol Evol.

[CR22] Lehninger AL (1970). Biochemistry.

[CR23] Lenckowski J, Walczak K (2007). Simplifying amino acid alphabets using a genetic algorithm and sequence alignment. Evolute Biol.

[CR24] Li T, Fan K, Wang J, Wang W (2003). Reduction of protein sequence complexity by residue grouping. Protein Eng.

[CR25] Liao S-M, Du Q-S, Meng J-Z, Pang Z-W, Huang R-B (2013). The multiple roles of histidine in protein interactions. Chem Cent J.

[CR26] Liu X, Liu D, Qi J, Zheng WM (2002). Simplified amino acid alphabets based on deviation of conditional probability from random background. Phys Rev E.

[CR27] Livingstone CD, Barton GJ (1993). Protein sequence alignments: a strategy for the hierarchical analysis of residue conservation. CABIOS.

[CR28] Lucchese G, Sinha AA, Kanduc D (2012). How a single amino acid change may alter the immunological information of a peptide. Front Biosci.

[CR29] Mahler HR, Cordes EH (1966). Biological chemistry.

[CR30] Maiorov VN, Crippen GM (1992). Contact potential that recognizes the correct folding of globular proteins. J Mol Biol.

[CR31] Mantel N (1967). The detection of disease clustering and a generalized regression approach. Cancer Res.

[CR32] Melo F, Marti-Renom MA (2006). Accuracy of sequence alignment and fold assessment using reduced amino acid alphabets. Proteins.

[CR33] Mirny LA, Shakhnovich EI (1999). Universally conserved positions in protein folds: reading evolutionary signals about stability, folding kinetics and function. J Mol Biol.

[CR34] Morlini I, Zani S (2012). Dissimilarity and similarity measures for comparing dendrograms and their applications. Adv Data Anal Classif.

[CR35] Murphy LR, Wallqvist A, Levy RM (2000). Simplified amino acid alphabets for protein fold recognition and implications for folding. Protein Eng.

[CR36] Muskal SM, Holbrook SR, Kim S-H (1990). Prediction of the disulfide-bonding state of cysteine in proteins. Protein Eng.

[CR37] Noivirt-Brik O, Hazan G, Unger R, Ofran Y (2013). Non local residue–residue contacts in proteins are more conserved than local ones. Bioinformatics.

[CR38] Peterson EL, Kondev J, Theriot JA, Phillips R (2009). Reduced amino acid alphabets exhibit an improved sensitivity and selectivity in fold assignment. Bioinformatics.

[CR39] Prlic A, Domingues FS, Sippl MJ (2000). Structure-derived substitution matrices for alignment of distantly related sequences. Protein Eng.

[CR40] Riddle DS (1997). Functional rapidly folding proteins from simplified amino acid sequences. Nat Struct Biol.

[CR41] Risler JL, Delorme MO, Delacroix H, Henaut A (1988). Amino acid substitutions in structurally related proteins. A pattern recognition approach. Determination of a new and efficient scoring matrix. J Mol Biol.

[CR42] Robson B, Suzuki E (1976). Conformational properties of amino acid residues in globular proteins. J Mol Biol.

[CR43] Rogov SI, Nekrasov AN (2001). A numerical measure of amino acid residues similarity based on the analysis of their surroundings in natural protein sequences. Protein Eng.

[CR44] Solis AD, Rackovsky S (2000). Optimized representations and maximal information in proteins. Proteins.

[CR45] Susko E, Roger AJ (2007). On reduced amino acid alphabets for phylogenetic inference. Mol Biol Evol.

[CR46] Tamura K (2011). MEGA5: molecular evolutionary genetics analysis using maximum likelihood, evolutionary distance, and maximum parsimony methods. Mol Biol Evol.

[CR47] Taylor WR (1986). The classification of amino acid conservation. J Theor Biol.

[CR48] Thomas PD, Dill KA (1996). An iterative method for extracting energy-like quantities from protein structures. Proc Natl Acad Sci USA.

[CR49] Wang J, Wang W (1999). A computational approach to simplifying the protein folding alphabet. Nat Struct Biol.

[CR50] Weathers EA, Paulaitis ME, Woolf TB, Hoh JH (2004). Reduced amino acid alphabet is sufficient to accurately recognize intrinsically disordered protein. FEBS Lett.

[CR51] Yampolsky LY, Stolzfus A (2005). The exchangeability of amino acids in proteins. Genetics.

[CR52] Zuo YC, Li QZ (2009). Using reduced amino acid composition to predict defense in family and subfamily: integrating similarity measure and structural alphabet. Peptides.

