# Supplementary material for: Unearthing the Root of Amino Acid Similarity
Source: J Mol Evol. 2013 Oct 1;77(4):159–69. doi: 10.1007/s00239-013-9565-0 (PMC6763418; doi:10.1007/s00239-013-9565-0)
Supplement: Supplementary file 1 — Supplementary material 1 (PDF 236 kb) [file 239_2013_9565_MOESM1_ESM.pdf]

Unearthing the root of Amino Acid Similarity

James D. Stephenson, Stephen J. Freeland

Supplementray Material

	Mahler	Lehninger	Robson	Dayhoff	Dickerson	Taylor	Risler	Crippen	Maiorov	Thomas	Riddle	Mirny	Wang	Prilic_SDM12	Prilic_HDSM17	Solis(G)	Solis(D)	Murphy	Rogov	Cieplak	Liu	Cannata	Fan	Li	EdgarSe-B	EdgarSe-V	Kosiol	Weathers	Andersen	Melo	Lenckowski	Etchebest	Solis_GBMR4	Zuo
Mahler																																		
Lehninger	0.248																																	
Robson	0.148	0.216																																
Dayhoff	0.116	0.258	0.148																															
Dickerson	0.300	0.295	0.248	0.185																														
Taylor	0.074	0.248	0.148	0.095	0.248																													
Risler	0.106	0.279	0.116	0.137	0.290	0.085																												
Crippen	0.311	0.358	0.269	0.311	0.337	0.300	0.258																											
Maiorov	0.169	0.290	0.158	0.169	0.258	0.190	0.158	0.216																										
Thomas	0.237	0.200	0.185	0.227	0.327	0.258	0.258	0.348	0.269																									
Riddle	0.227	0.285	0.174	0.227	0.306	0.227	0.195	0.327	0.216	0.200																								
Mirny	0.116	0.237	0.116	0.158	0.332	0.148	0.148	0.300	0.158	0.195	0.185																							
Wang	0.227	0.285	0.174	0.227	0.306	0.227	0.195	0.327	0.216	0.200	0.000	0.185																						
Prilic_SDM12	0.111	0.243	0.079	0.122	0.274	0.100	0.058	0.264	0.164	0.222	0.200	0.111	0.200																					
Prilic_HDSM17	0.095	0.269	0.106	0.137	0.290	0.074	0.043	0.258	0.158	0.258	0.216	0.148	0.216	0.048																				
Solis(G)	0.311	0.337	0.279	0.343	0.422	0.322	0.269	0.422	0.343	0.327	0.327	0.322	0.327	0.264	0.269																			
Solis(D)	0.158	0.311	0.148	0.169	0.290	0.137	0.063	0.290	0.179	0.269	0.216	0.179	0.216	0.100	0.074	0.279																		
Murphy	0.090	0.253	0.100	0.058	0.243	0.090	0.079	0.285	0.111	0.222	0.190	0.111	0.190	0.063	0.079	0.285	0.122																	
Rogov	0.179	0.311	0.148	0.179	0.279	0.158	0.116	0.311	0.211	0.258	0.206	0.211	0.206	0.122	0.116	0.300	0.095	0.143																
Cieplak	0.306	0.353	0.243	0.253	0.332	0.306	0.285	0.300	0.285	0.269	0.258	0.285	0.258	0.269	0.274	0.458	0.264	0.248	0.264															
Liu	0.258	0.211	0.153	0.216	0.243	0.237	0.227	0.274	0.216	0.137	0.264	0.206	0.264	0.190	0.216	0.390	0.248	0.211	0.258	0.269														
Cannata	0.095	0.258	0.095	0.127	0.290	0.085	0.022	0.258	0.137	0.248	0.216	0.137	0.216	0.048	0.032	0.279	0.074	0.069	0.127	0.274	0.206													
Fan	0.100	0.285	0.132	0.090	0.264	0.079	0.058	0.285	0.143	0.243	0.211	0.143	0.211	0.085	0.079	0.306	0.111	0.053	0.132	0.279	0.232	0.048												
Li	0.095	0.279	0.127	0.127	0.269	0.074	0.032	0.279	0.158	0.237	0.195	0.127	0.195	0.079	0.074	0.290	0.095	0.069	0.137	0.285	0.216	0.043	0.037											
EdgarSe-B	0.116	0.269	0.106	0.085	0.258	0.095	0.063	0.290	0.137	0.248	0.206	0.127	0.206	0.048	0.063	0.279	0.106	0.048	0.106	0.285	0.216	0.053	0.058	0.085										
EdgarSe-V	0.116	0.269	0.116	0.085	0.237	0.095	0.053	0.279	0.148	0.248	0.185	0.137	0.185	0.058	0.074	0.279	0.106	0.048	0.106	0.274	0.216	0.063	0.069	0.063	0.032									
Kosiol	0.374	0.443	0.322	0.258	0.306	0.353	0.353	0.337	0.395	0.337	0.379	0.374	0.379	0.316	0.364	0.506	0.332	0.316	0.311	0.237	0.306	0.364	0.337	0.353	0.322	0.300								
Weathers	0.206	0.295	0.206	0.216	0.264	0.185	0.216	0.327	0.269	0.253	0.211	0.227	0.211	0.200	0.206	0.369	0.216	0.200	0.206	0.300	0.306	0.216	0.211	0.185	0.227	0.206	0.358							
Andersen	0.100	0.274	0.111	0.132	0.295	0.079	0.016	0.264	0.164	0.243	0.179	0.132	0.179	0.074	0.058	0.274	0.069	0.074	0.122	0.279	0.232	0.037	0.053	0.027	0.079	0.069	0.358	0.211						
Melo	0.295	0.343	0.211	0.243	0.300	0.295	0.274	0.322	0.295	0.227	0.195	0.285	0.195	0.237	0.285	0.374	0.264	0.216	0.211	0.179	0.300	0.285	0.269	0.264	0.264	0.243	0.227	0.227	0.258					
Lenckowski	0.227	0.379	0.237	0.269	0.348	0.216	0.174	0.348	0.248	0.379	0.348	0.269	0.348	0.211	0.174	0.358	0.185	0.222	0.237	0.364	0.316	0.174	0.200	0.185	0.216	0.227	0.432	0.306	0.179	0.374				
Etchebest	0.127	0.290	0.158	0.148	0.279	0.116	0.053	0.290	0.169	0.258	0.227	0.158	0.227	0.090	0.085	0.258	0.063	0.090	0.127	0.295	0.269	0.074	0.079	0.063	0.095	0.095	0.353	0.216	0.048	0.264	0.185			
Solis_GBMR4	0.369	0.406	0.274	0.327	0.364	0.369	0.348	0.385	0.306	0.343	0.185	0.285	0.185	0.311	0.358	0.406	0.337	0.290	0.243	0.285	0.395	0.358	0.322	0.337	0.306	0.295	0.311	0.300	0.332	0.169	0.427	0.337		
Zuo	0.100	0.295	0.132	0.132	0.295	0.079	0.027	0.274	0.153	0.253	0.200	0.153	0.200	0.085	0.058	0.285	0.069	0.074	0.122	0.279	0.243	0.048	0.063	0.037	0.079	0.069	0.358	0.200	0.022	0.269	0.169	0.037	0.343	

Table S1: Distance matrix showing the dissimilarity of 34 simplified amino acid alphabets.

The values shown are distances between alphabets, calculated by subtracting the similarity measurements from 1, therefore a value of 0 represents identical alphabets (Riddle and Wang) and 1 would represent entirely different alphabet simplifications.
